# QTL mapping for bacterial wilt resistance in peanut (*Arachis hypogaea* L.)

**DOI:** 10.1007/s11032-015-0432-0

**Published:** 2016-01-30

**Authors:** Yongli Zhao, Chong Zhang, Hua Chen, Mei Yuan, Rick Nipper, C. S. Prakash, Weijian Zhuang, Guohao He

**Affiliations:** Tuskegee University, Tuskegee, AL 36088 USA; Key Laboratory of Crop Molecular and Cell Biology, Fujian Agriculture and Forestry University, Fuzhou, China; Shandong Peanut Research Institute, Qingdao, China; Floragenex Inc., Portland, OR 97239 USA

**Keywords:** QTL analysis, Bacterial wilt, SNP, SSR, RAD sequencing, BSA, Resistance gene homolog, Peanut

## Abstract

Bacterial wilt (BW) caused by *Ralstonia solanacearum* is a serious, global, disease of peanut (*Arachis hypogaea* L.), but it is especially destructive in China. Identification of DNA markers linked to the resistance to this disease will help peanut breeders efficiently develop resistant cultivars through molecular breeding. A F_2_ population, from a cross between disease-resistant and disease-susceptible cultivars, was used to detect quantitative trait loci (QTL) associated with the resistance to this disease in the cultivated peanut. Genome-wide SNPs were identified from restriction-site-associated DNA sequencing tags using next-generation DNA sequencing technology. SNPs linked to disease resistance were determined in two bulks of 30 resistant and 30 susceptible plants along with two parental plants using bulk segregant analysis. Polymorphic SSR and SNP markers were utilized for construction of a linkage map and for performing the QTL analysis, and a moderately dense linkage map was constructed in the F_2_ population. Two QTL (*qBW*-1 and *qBW*-2) detected for resistance to BW disease were located in the linkage groups LG1 and LG10 and account for 21 and 12 % of the bacterial wilt phenotypic variance. To confirm these QTL, the F_8_ RIL population with 223 plants was utilized for genotyping and phenotyping plants by year and location as compared to the F_2_ population. The QTL *qBW*-1 was consistent in the location of LG1 in the F_8_ population though the QTL *qBW*-2 could not be clarified due to fewer markers used and mapped in LG10. The QTL *qBW*-1, including four linked SNP markers and one SSR marker within 14.4-cM interval in the F_8_, was closely related to a disease resistance gene homolog and was considered as a candidate gene for resistance to BW. QTL identified in this study would be useful to conduct marker-assisted selection and may permit cloning of resistance genes. Our study shows that bulk segregant analysis of genome-wide SNPs is a useful approach to expedite the identification of genetic markers linked to disease resistance traits in the allotetraploidy species peanut.

**Electronic supplementary material:**

The online version of this article (doi:10.1007/s11032-015-0432-0) contains supplementary material, which is available to authorized users.

## Introduction

Bacterial wilt (BW), caused by *Ralstonia solanacearum,* is a disease of considerable global importance. It was first recorded in South Africa during 1924–1925 in the coastal belt of Natalby (McClean 1930). The pathogen is primarily dependent on the moisture-holding capacity of the soil for its existence. This soilborne pathogen infects the plant roots through wounds and spreads rapidly via the vascular system (Kelman and Sequeira 1965; Schmit 1978; Vasse et al. 1995). Bacterial wilt is one of the most prevalent plant bacterial diseases, affecting more than 450 plant species including peanut, and is primarily distributed across tropical and subtropical humid countries (Buddenhagen 1986; Wicker et al. 2007). In China, BW affects 10–30 % of the peanut production area, may cause significant economic loss, and may even lead to total crop failure in the extreme instances (Yu et al. 2011). BW is caused by a soilborne pathogen, so it is challenging to control its spread and limit its damaging effects. Conventional management strategies of BW such as crop rotation, adjusting the date of planting, cultural methods, and soil treatment are not very effective, especially because of the broad host range of this pathogen (Cao et al. 2009). Although BW disease could be controlled by applying fertilizers and soil amendments to change soil pH and reduce survival and activity of the pathogen (Lu et al. 2010), the most effective and preferred strategy is to develop resistant cultivars.

Improving the BW resistance is one of the major objectives for peanut breeders in China and many other countries including Indonesia, Vietnam, and Uganda (Liao 2014). Conventional breeding for disease resistance has attempted to address the issue of BW disease in the past, and several resistant cultivars have been developed and used in peanut production (Yu et al. 2011). However, the source of resistance to BW used in such peanut breeding is limited to a few lines (Liao 2014). Furthermore, the resistance to BW disease is inversely proportional to yield and seed quality (Lu et al. 2010), making it difficult to combine these important traits into a single cultivar. To locate new sources of resistance lines, Lu et al. (2010) recently evaluated the resistance to BW disease in the peanut mini core collection from ICRISAT in India and reported that high resistance to BW was found in two genotypes (ICG9249 and ICG1262523), which were genetically different from those resistant lines used traditionally for breeding in China. Clearly, use of such new resistance lines would broaden the genetic base of future peanut cultivars, thus providing greater stability of disease resistance.

The genetic basis of BW resistance in peanut is not well understood. Liao et al. (1986) observed that a cytoplasmic effect was associated with the BW resistance in the dragon line landraces, but the mechanism of the cytoplasmic effect on the resistance was unclear. However, this type of association was not found in the reciprocal crosses where Spanish and Valencia types were involved. They also suggested that both additive and dominant genes might play a role in the inheritance of resistance because high significant variances of general combining ability (GCA) and special combining ability (SCA) were detected (Shan et al. 1998). Although quantitative inheritance was displayed in the RIL population, Ren et al. (2008) suggested that there were two major genes related with the BW resistance.

Molecular breeding through marker-assisted selection not only accelerates the breeding of crops, but also facilitates pyramiding multiple genes into a single cultivar. Many efforts have been made to identify molecular markers linked to the BW resistance for molecular breeding in peanut. Jiang et al. (2007) identified two flanking SSR markers related to the resistance gene at a distance of 10.9 and 13.8 cM. A similar study using AFLP markers identified additional two flanking markers linked to the resistance gene with a distance of 8.12 and 11.46 cM (Ren et al. 2008). Differential expression was also used to detect transcript-derived fragments (TDFs) associated with the resistance to BW (Peng et al. 2011; Ding et al. 2012). However, current information on markers tightly associated with the resistant trait remains scant, limiting the use of marker-assisted selection in the resistance breeding against this disease. To identify the tightly linked markers, cosegregation of molecular markers with the resistant trait in a mapping population is vital, which depends on (1) whether the mapping population has enough progenies to display the targeted recombinants and (2) whether markers are distributed genome-wide allowing investigators to detect the recombination.

Genome-wide marker analysis in plant population is useful in investigating the genetic architecture underpinning quantitative and other phenotypic traits (Davey and Blaxter 2011). Restriction-site-associated DNA sequencing (RAD-seq) is a commonly used approach where DNA adjacent to each instance of a restriction enzyme recognition site is sequenced using next-generation DNA sequencing (NGS) platform (Baird et al. 2008). NGS systems enable the generation of massive amounts of DNA sequence information and thus facilitate rapid discovery of thousands of SNPs across a target genome.

Bulk segregant analysis (BSA) has traditionally been employed to locate markers linked to any specific gene or genomic region (Michelmore et al. 1991). BSA has successfully identified markers associated with a variety of traits in many different plant species (Quarrie et al. 1999; Brauer et al. 2006; Wenger et al. 2012; Becker et al. 2012). Combing these two powerful approaches, BSA and RAD-SNPs, may enable rapid detection of SNPs linked to the gene of interest. There are no DNA markers identified in cultivated peanut linked to any disease resistance gene so far with the exception of a marker for the root-knot nematode resistance gene in a wild *Arachis* species (Nagy et al. 2010). Therefore, this study aimed to (1) use RAD-seq technology in combination with the BSA method to identify SNP markers linked to the BW resistance in peanut, and (2) perform QTL mapping for resistance to BW disease using genotyping and phenotyping data in the F_2_ and F_8_ populations derived from different years and locations.

## Materials and methods

### Plant materials and bacterial inoculations

Two cultivated peanut cultivars, Yueyou 92 and Xinhuixiaoli, were used as parental genotypes to generate the mapping population. Cultivar Yueyou 92 is highly resistant to BW disease, while cv. Xinhuixiaoli is highly susceptible to BW. A total of one hundred thirty F_2_ plants were produced and grown in the field at Fujian Agriculture University, China. Plants in the F_2_ population were inoculated with *Ralstonia solanacearum* virulent strain Rs-P.362200 using a suspension of 1 × 10^8^ strains per milliliter 35 days before harvest. Five additional F_2_ plants derived from the same cross (Yueyou 92 × Xinhuixiaoli) were inoculated with water as a control. Two leaflets from each of five leaves in individual plant were cut and inoculated using the scissors soaked in suspension. This highly effective, leaf-cutting method was previously described by Zhang et al. (2010). At 15, 25, and 35 days after inoculation (dai), disease symptoms were scored using the following 1–4 scale: 1, resistant to BW, no wilting or wilting only presents in some cut leaves (designated as R); 2, moderate resistant, wilting presents in the uncut leaves and stem of the inoculated branches (MR); 3, moderate susceptible, wilting presents in the leaves and stems of branches without inoculated (MS); and 4, susceptible, wilting presents at the whole plant or whole plant death (S). Eighty plants from the resistant and susceptible parents were also inoculated with water as controls. To confirm QTL identified in the F_2_ population, two hundred twenty-three F_8_ recombinant inbreeding lines (RILs) advanced from the F_2_ population (Yueyou 92 × Xinhuixiaoli) were also inoculated and phenotypic data were obtained at 27 dai for QTL analysis in the F_8_ population.

### DNA extraction

Genomic DNAs were extracted from fresh leaf tissue of 130 F_2_ individual plants and 223 F_8_ RIL plants using the CTAB method with minor modification (Murray and Thompson 1980). Leaf tissue was ground in liquid nitrogen. CTAB extraction solution was added and incubated at 65 °C for 15–30 min. The same volume of chloroform–IAA (24:1) was added, shaken, and centrifuged for 10 min at 10,000 rpm. The supernatant was transferred to a fresh tube. A precipitate was formed by adding an equal volume of isopropanol. The tube was centrifuged again, the resulting supernatant was discarded, and the pellet was washed with 75 % ETOH. The DNA sample was resuspended in H_2_O and RNase. DNA quality and quantity were determined by agarose gel electrophoresis and spectrophotometer analysis.

### Production of RAD libraries

The 30 most resistant (score 1) and 30 most susceptible individuals (score 4) were collected from the F_6_ RIL population developed from the F_2_ population of the same cross between Yueyou 92 and Xinhuixiaoli, and genomic DNA was extracted from each individual plant. An equal amount of DNA from each of the resistant plants was bulked to form a resistant DNA pool, and the same procedure was used to generate a susceptible DNA pool. These two DNA pools and two parental DNA samples (resistant vs. susceptible) were used to prepare RAD libraries for DNA sequencing at Floragenex (Eugene, OR) and processed into RAD libraries similar to the method of Baird et al. 2008. Briefly, 1000 ng of genomic DNA was digested for 60 min at 37 °C in a 50-μL reaction with 100 units (U) of *PstI* (New England Biolabs, MA). After digestion, samples were heat-inactivated for 20 min at 80 °C followed by addition of P1 adapter(s), a modified Illumina adapter (Illumina, CA). *PstI* P1 adapters each contained a unique multiplex sequence index (barcode) which was read during the first 10 nucleotides of the Illumina sequence read. One microliter 10 μM P1 adapters was added to each sample along with 6 μL 10× NEB T4 DNA ligase buffer, 1.0 μL (1000 U) T4 DNA ligase (high concentration, Enzymatics, Inc), and 1 μL Qiagen buffer EB (Qiagen, CA), which was then incubated at room temperature (RT) for 1 h. Samples were again heat-inactivated for 10 min at 65 °C, pooled, and randomly sheared with a Bioruptor (Diagenode, NJ) to an average size of 500 bp. Samples were then run out on a 1.5 % agarose (Sigma, MO), 0.5× TBE gel, and DNA 200–800 bp was isolated using a MinElute Gel Extraction Kit (Qiagen, CA). End blunting enzymes (Enzymatics, MA) were then used to polish the ends of the DNA. Samples were purified using a MinElute column (Qiagen, CA) and 15 U of Klenow exo^−^(Enzymatics, MA) was used to add adenine (Fermentas, NY) overhangs on the 3′ end of the DNA at 37 °C. After subsequent purification, 1 μL of 1 μM P2 adapter, a divergent modified Illumina adapter (Illumina, CA), was ligated to the DNA fragments at room temperature (RT). Samples were again purified and eluted in 15 μL. The eluate was quantified using a Qubit fluorimeter, and 10 ng of this product was used in PCR amplification with 25 μL Phusion Master Mix (NEB, MA), 5 μL of 10 μM modified Illumina amplification primer mix (Illumina, CA), and 19 μL H_2_O. Phusion PCR settings followed product guidelines for a total of 18 cycles. Again, samples were gel-purified, excising DNA from the 300- to 700-bp-size range, and diluted to 10 nM.

### Illumina sequencing

A set of RAD libraries generated from the above pools was run on an Illumina HiSeq 2000 at the Oregon State University Center for Genome Research and Biocomputing High in Corvallis, Oregon. Standard Illumina protocols were followed for a 2 × 100 bp paired end sequencing run.

### Bioinformatic identification of SNPs related to resistance to BW disease

Variant calling and SNP identification were performed using the strategies outlined in Pegadaraju, et al. (2013). Briefly, a de novo reference assembly was constructed from the resistant parent using Velvet (Zerbino and Birney 2008), which served as a scaffold for sequence alignment. 98,685 contigs were constructed, covering approximately 40 megabase pairs of the *Arachis* genome. Sequence reads from all samples were aligned to the reference using Bowtie and variants called using SAMtools (Langmead et al. 2009; Li et al. 2009). After variant calling, a VCF file cataloging all putative variants was parsed using a custom Perl script to identify those alleles enriched in the susceptible bulk (AF ≥ 0.60) and less abundant in the resistant bulk (AF ≤ 0.50). This mapping approach was implemented due to the recessive nature of susceptibility traits in the population and the absence of any clearly linked markers when attempting to identify variants associated with the resistance genes.

### Construction of genetic linkage map

In our previous study, 14.5 % of a total of 9274 simple sequence repeats (SSRs) showed polymorphism within peanut germplasm (Zhao et al. 2012). The polymorphic 1343 SSR markers were utilized for genotyping the F_2_ progenies to develop a linkage map. SSR markers are advantageous for linkage mapping due to the ease of scoring, high reproducibility, multiallelic variation, and codominant mode of inheritance. The PCR program included 94 °C/3 min for initial denaturation, followed by 35 cycles of 94 °C/30 s, 55 °C/30 s, and 72 °C/30 s, and 72 °C/5 min for final extension. PCR products were resolved in polyacrylamide gel in LI-COR 4300 DNA Analyzer (LI-COR, NA). The SNPs linked to the resistance were subjected for SNP genotyping in the F_2_ population with KASP procedure by LGC Genomics (Beverly, MA).

Linkage analyses were performed using JoinMap 4 software (Van Ooijen and Voorrips 2006). The Kosambi mapping function was used to transform the recombination frequency to genetic distances (Kosambi 1944). Marker order was assigned using the regression mapping algorithm with maximum recombination frequency of 0.4. Linkage groups were identified using minimum logarithm of odds (LOD) values of 4. The segregation ratio at each marker locus was statistically analyzed against the expected Mendelian segregation ratios by *χ*^2^ tests.

### QTL analysis of BW-resistant trait

Genotyping data and phenotyping data for BW resistance obtained in F_2_ and F_8_ populations were used for QTL analysis. The composite interval mapping (CIM) (Zeng 1994) using WinQTLCart 2.5 (Wang et al. 2007) was performed to identify QTL-related markers with Model 6 and backward regression method. To achieve normally distributed trait data, disease severity values were evaluated and transformed to log_10_ for QTL analysis. To obtain more precise results, the walk speed was 1 cM. A LOD score of 3 was used as the threshold for testing significance of QTL peaks with 1000 permutations and significance level of *P* ≤ 0.05. The proportion of the total phenotypic variance explained by each QTL was calculated as an *R*^2^ value. The software package R/qtl (Broman et al. 2003) was also used to verify the QTL. Single QTL analysis was performed using Haley-Knott regression method, and 95 % Bayes interval was used to obtain interval estimates of QTL location.

## Results

### Evaluation of bacterial wilt resistance trait

**Fig. 1:**
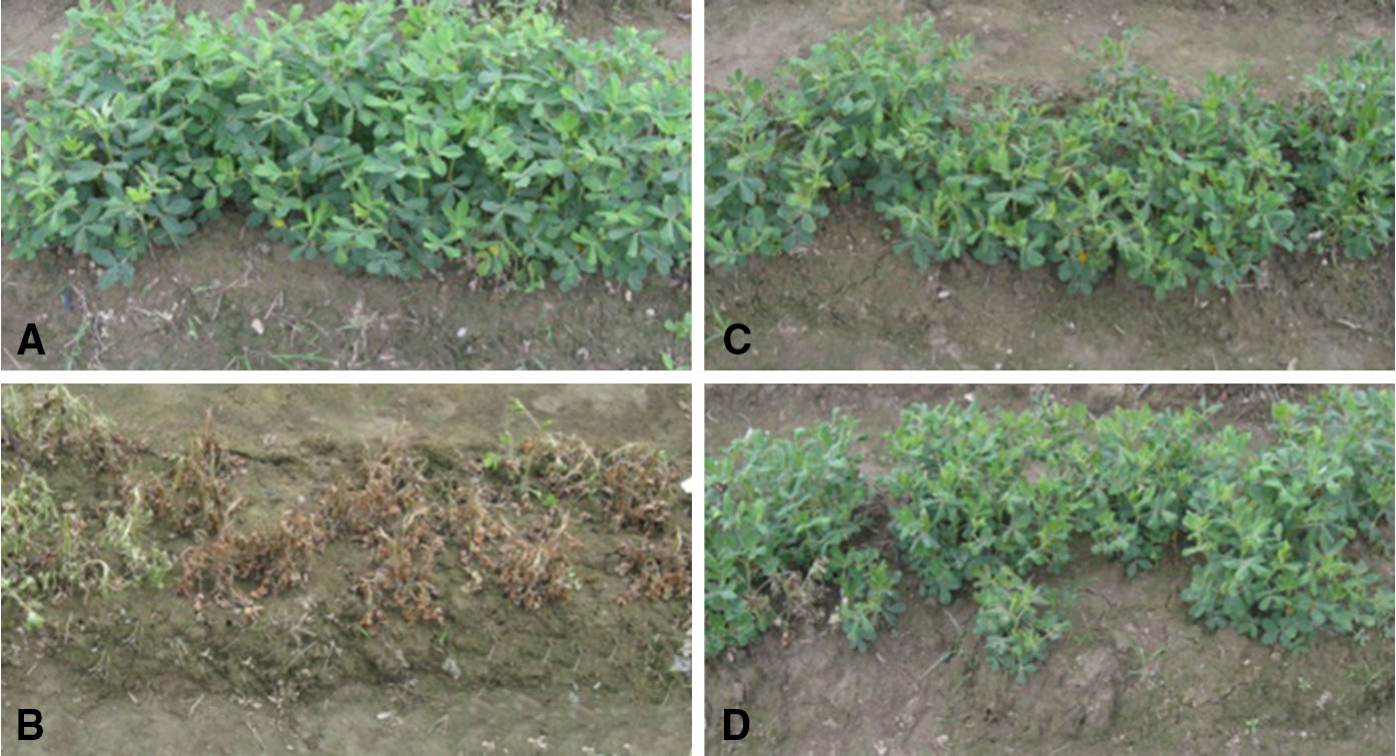
Phenotypes of resistant and susceptible parents with or without inoculation of *R. solanacearum*. **a** Phenotype of susceptible parent Xinhuixiaoli without inoculation of Rs. **b** Phenotype of susceptible parent Xinhuixiaoli inoculated with Rs for 15 days. **c** Phenotype of resistance parent without inoculation of Rs. **d** Phenotype of resistance parent inoculated with Rs for 15 days

A highly virulent strain of *R. solanacearum* Rs-, viz P.362200, was used to evaluate the resistance to bacterial wilt in the cultivated peanut by the leaf-cutting method. Two parental lines, Yueyou 92 and Xinhuixiaoli, clearly displayed differential reactions to the inoculation with the pathogen. Loss of leaf color or yellowing of leaves was observed in Xinhuixiaoli within a few dai. Wilt symptoms developed rapidly in the cut leaves, spread to uncut leaves, and subsequently spread to leaves of other branches, leading to whole plant wilt or death in 15–25 dai. Yueyou 92 showed no apparent symptoms or very little wilt in the cut leaves (Fig. 1). The control parental plants, inoculated with water, showed the normal phenotype throughout the study.

Genetic analysis of resistance was performed in the F_1_ and F_2_ populations. F_1_ plants were susceptible, with a score of 4 after inoculation with strain Rs-P.362200, indicating that the resistance to bacterial wilt strain Rs-P.362200 is controlled by recessive gene(s) in peanut. In the F_2_ population, 64 out of 130 inoculated plants were completely resistant (disease score 1, R), 16 were moderately resistant (score 2, MR), 23 were moderately susceptible (score 3, MS), and 27 were fully susceptible (score 4, S) at 15 dai. The symptom scores (1–4) were recorded as 41 (R), 16 (MR), 35 (MS), and 38 (S) at the 25 dai, and 30 (R), 14 (MR), 16 (MS), and 69 (S) at 35 dai. The number of plants with resistance traits decreased over time, while the number of plants with susceptible symptoms increased as the number of dai increased. The full range of disease symptoms was evident in the susceptible parent Xinhuixiaoli at 25 dai, so the phenotypic data of F_2_ individuals at 25 dai was employed for further analysis including QTL analysis. To test whether the resistance trait is controlled by a single gene, phenotypic data of moderate resistant, moderate susceptible, and susceptible plants, as long as susceptible symptoms persisted, were considered as susceptible data (disease score 2–4) versus resistance data (disease score 1). Based on the *χ*^2^ test, the null hypothesis of the ratio of susceptible to resistant data fitting into 3:1 segregation was accepted at 25 and 35 dai, though it was rejected with the data at 15 dai (Table 1). In our preliminary study, a linkage map was constructed using polymorphic SSR markers to test whether there were any DNA markers linked to the resistant trait. As a result, no SSR markers were found related to the resistant trait in this SSR-based linkage map.

**Table 1:** Chi-square test for 3:1 segregation ratio of the BW-susceptible versus BW-resistant phenotypes at different days after inoculation in the F_2_ population

Days after inoculation (dai)	*χ* ^2^	*P* value, df = 1
15	40.7	*P* < 0.001
25	2.96	0.10 < *P* < 0.05
35	0.21	0.90 < *P* < 0.50

### Identification of SNPs related to the resistance to BW

To rapidly identify trait-related DNA markers, two parental DNA samples and two bulked DNA samples (resistant vs. susceptible) were subjected to the bulked segregant analysis using SNPs derived from the next-generation sequencing RAD-seq technology. A total of 17,000 SNPs were discovered from over 80 million “100 base reads” RAD-seqs in these four samples. Among the identified SNPs, 180 were identified as putative SNPs related to the bacterial wilt-resistant trait by the BSA method. However, only 26 out of 180 SNPs showed allelic variation among 130 F_2_ individuals and 223 F_8_ plants using the KASP SNP genotyping method (LGC Genomics, MA), and the remaining SNPs displayed homeologous variation (between two subgenomes), which was abundant in the allotetraploidy species of the cultivated peanut.

### Construction of genetic linkage map

In our earlier study, 1343 polymorphic SSR markers were identified with a panel of peanut germplasm (Zhao et al. 2012). However, the number of polymorphic SSR markers was only 309 in the biparental F_2_ population (Supplementary file, S1). Among these 309 polymorphic loci, 57 loci (18.7 %) were significantly deviated from the expected 1:2:1 or 3:1 segregation ratio at *P* ≤ 0.05. Construction of a linkage map using the polymorphic SSR and SNP markers mentioned above has resulted in all 20 linkage groups. A total of 237 markers were mapped, which covered 1627.4 cM with an average distance of 6.8 cM (Supplementary file, S2). Polymorphic SNPs were spread into eleven different linkage groups. The longest linkage group was 153.7 cM in LG1 with 23 marker loci. The shortest one was 30.3 cM in LG 17 having five loci. These genotyping data, combined with phenotyping data at 25 dai, were used for QTL mapping.

### Detection of QTL in the linkage map

**Fig. 2:**
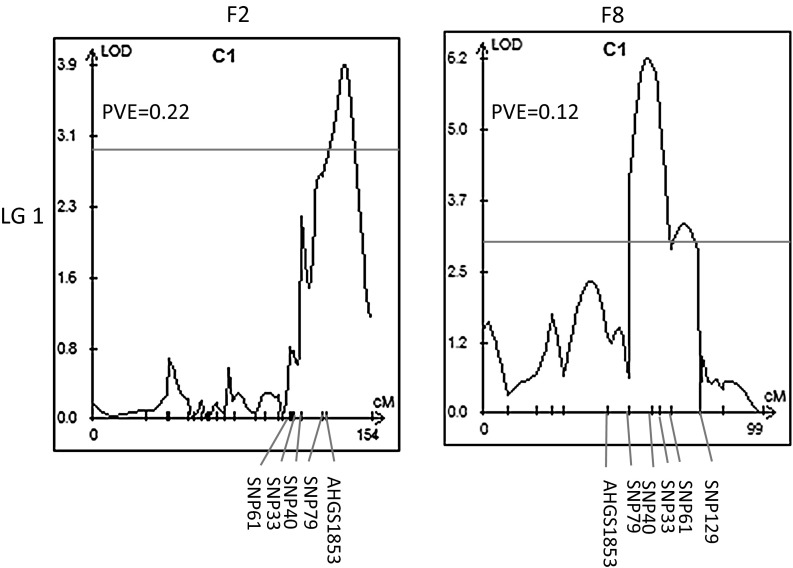
QTL *qbw*-1 detected in F2 and F8 populations using WinQTLCart

QTL analysis was performed for resistance phenotypic data using the CIM approach in the WinQTLCart 2.5 version. Two QTL associated with resistance to BW were detected in two genomic regions in LG1 and LG10 with LOD = 3.9 and 3.2, respectively. These two QTL (designated as *qBW*-1 and *qBW*-2) and their confidence interval, additive effect, and *R*^2^ are listed in Table 2. Two QTL *qBW*-1 and *qBW*-2 for resistance to BW had an additive effect of −0.15 and −0.11, and each explained 21 and 12 % of the phenotype variance, respectively. The R/qtl software was also used to confirm these two QTL. The intervals of QTL were detected and located on the same regions of LG 1 and LG 10. The function “find.marker” was used to identify the markers closest to the QTL peak. The result showed that SNP79 was the closest one to the *qBW*-1 in LG 1, but no marker was identified close to the *qBW*-2 in LG 10. Varshney et al. (2014) considered QTL as stable if they appeared in more than one location for the specified trait and QTL as consistent if they appear in more than 1 year/season for the specific trait (Varshney et al. 2014). Our F_2_ phenotypic data were collected from one location; therefore, the F_8_ RIL population advanced from this F_2_ population grew in a different year, and a different location was utilized to confirm the QTL identified in the F_2_ population. The phenotypic data of 223 individuals were obtained only at 27 dai. Flanking markers of two QTL and all putative trait-related SNPs were used to construct the F_8_ linkage map. A total of 74 markers were mapped into 10 groups. The QTL mapping with phenotypic data showed that the *qBW*-1 was clarified located in the LG 1 and the interval of the *qBW*-1 included SNP79 along with other four SNPs (Fig. 2). However, the *qBW*-2 was not confirmed in the F_8_ population, which might be due to fewer markers mapped in the LG 10 in the F_8_ population.

**Table 2:** QTL detected in F_2_ and F_8_ populations derived from the cross between two cultivars Yueyou 92 and Xinhuixiaoli in peanut

QTL	Linkage group	Flanking marker interval	LOD	Additive effect	Dominant effect	*R* ^2^
*qBW*-1	F_2_—LG1	SNP79–AHGS1853	3.911	−0.154	0.137	0.216
	F_8_—LG1	SNP79–SNP129	6.219	−0.056	−0.019	0.119
*qBW*-2	F_2_—LG10	Ai119F10–AHS3174	3.164	−0.111	0.158	0.120

**Fig. 3:**
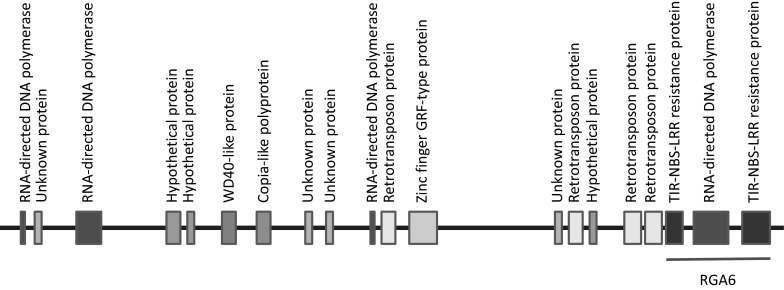
Schematic representation of BAC clone (HQ637177.1, GenBank accession number) containing disease resistance protein, which was named as RGA 6 by Ratnaparkhe et al. (2011)

The marker SNP79 was specifically located in the interval peak of QTL *qBW*-1 in the map of the F_2_ and F_8_ population (Fig. 2). The sequence of SNP79 was found located at a RNA-directed DNA polymerase near a TIR-NBS-LRR gene within a BAC clone (GenBank Accession Number HQ637177.1) by BLASTx against the National Center for Biotechnology Information (NCBI). The schematic representation of this BAC clone was based on the order of genes on the clone AHF-303L13 complete sequence (Fig. 3, Ratnaparkhe et al. 2011). Four other SNPs within QTL *qBW*-1 identified in F_8_ were not homologous to any genes.

## Discussion

The availability of molecular markers, particularly a large number of SSR markers, has made it possible to construct several framework linkage maps in the cultivated peanut (Ravi et al. 2011; Varshney et al. 2009; Qin et al. 2012; Wang et al. 2012). In the present study, 237 markers with average distances of 6.8 cM mapped in the linkage map of the F_2_ population were thought to be sufficient for QTL analysis even though the peanut has a large genome size (2800 Mbp), because the power of detecting a QTL was virtually the same for a marker spacing of 10 cM as for an infinite number of markers (Darvasi et al. 1993). We were able to detect two QTL for the resistance to BW disease in the F_2_ segregation population. Using a BLAST analysis, the marker SNP79 in the QTL *qBW*-1 showed a homology to the BAC clone containing resistance gene homologs (RGH) described by Ratnaparkhe et al. (2011). Disease resistance gene homologs have been identified and cloned from a variety of plant species including peanut (Bai et al. 2002; Pan et al. 2000; Hunger et al. 2003; Ratnaparkhe et al. 2011; Kang et al. 2012). Almost all homologs were genetically closely linked with known disease resistance loci in *Arabidopsis thaliana* (Aarts et al. 1998). Ratnaparkhe et al. (2011) have sequenced two peanut BAC clones, previously identified as showing strong hybridization signals with multiple R-gene probes and thus considered likely to contain clusters of R genes. As a result, they found five RGHs in a BAC clone and one RGH in another BAC clone. The QTL *qBW*-1 identified in this study, related to the BAC clone containing one RGH, could be considered as a candidate gene conferring resistance to BW in peanut. Polygenic resistance to bacterial wilt disease has been described in tomato (Thoquet et al. 1996) and in *A. thaliana* (Godiard et al. 2003). In peanut, Ren et al. (2008) reported that resistance to BW disease was controlled by two major genes on the basis of genetic recombination of two AFLP markers with the disease-resistant trait. Without a detailed genetic map, it is difficult to conclude whether these two major genes were located in the same chromosome or different chromosomes. Further, the resistance gene exhibited dominance or partial dominance effects in the study of Ren et al. (2008). In our study, the resistance was recessive, indicating that resistant genes from our study versus the study of Ren et al. (2008) are different. Therefore, two QTL detected in this study are not comparable to their two major genes. Nevertheless, the putative resistance-related markers identified in this study would facilitate the further discovery and cloning of disease resistance genes for bacterial wilt in peanut.

We have identified more than 17,000 genome-wide SNPs by next-generation sequencing RAD-seqs and screened these SNPs to detect an association between SNP and the resistance to BW by bulk segregant analysis. A much higher proportion of SNPs showed homeologous variation rather than allelic variation. An appropriate SNP calling pipeline should improve the SNP discovery in allotetraploid peanut. One of 26 trait-linked SNPs was found to be in the interval of QTL *qBW*-1, suggesting that it could be a true QTL for resistance to BW. Furthermore, this SNP was located in the TIR-NBS-LRR disease resistance gene that shared a high degree of similarity to three genes, *Phaseolus* CMR1 (ABH07384.1), *Medicago* TIR (Mt7g087890.1), and *Lens* (CAD56833.1) (Ratnaparkhe et al. 2011). Seo et al. (2006) reported that the *Phaseolus* CMR1 conferred resistance to gemini virus. To determine the molecular nature of this TIR-NBS-LRR gene in peanut, we have cloned the full length of the gene from the resistant parent and found down-regulated genes induced by *R. solanacearum* strain Rs-P.362200, based on our microarray analysis (unpublished data). Coincidentally, in the present study, the linked SNP79 within the interval of QTL *qBW*-1 and its similarity with a disease resistance gene provides the potential application of marker-assisted selection in peanut resistance breeding for BW disease. The *qBW*-2 was identified in the LG 10 in the F_2_ population but could not be confirmed in the F_8_ population. Further study should identify more markers in the genetic linkage map of the F_8_ population to ensure QTL *qBW*-2.

The QTL *qBW*-1 in the F_2_ population contained two markers, SNP79 and AHGS1853, although three trait-related SNP33, SNP40, and SNP61 were close by. While these SNPs and SNP79 resided in the QTL region with significant LOD = 6.2 and within 14.4 cM interval in F_8_ population, they were located in the chromosome 2 through the BLAST analysis in the web of peanut base (http://www.peanutbase.org). QTL analysis in different generations revealed a slight bias in the estimates of QTL, and this may be because of the confounding effect of the population size and the marker numbers. The early generation is often of insufficient population size to warrant a high QTL detection power (Wurschum 2012). In this study, 130 individuals were in the F_2_ population while 223 in the F_8_ population. Four trait-related SNPs fit in one QTL in the F_8_ population and may be attributed to the high number of individuals (>200) in the segregation population to detect a reliable QTL. On the other hand, employing enough markers to detect the existing recombination in the segregation population could be also important in the QTL analysis. In the present study, the QTL *qBW*-2 identified in the F_2_ population could not be confirmed in the F_8_ population, which might be due to the fewer markers mapped in LG 10. Without a high-resolution map, it is difficult to identify tightly linked markers because recombination can occur between a marker and QTL, and reduce the reliability and usefulness of the marker (Collard et al. 2005). We have generated a large number of SNPs from RAD-tags. However, abundant homeologous variation was observed in the allotetraploid peanut. A bioinformatic tool is critical to effectively distinguish between allelic polymorphisms (between accessions) and homeologous variation (between subgenomes). Therefore, genotyping by sequencing (GBS) could be used to reliable markers <1 cM away from the gene for marker-assisted selection (Michelmore 1995).

Because of the paucity of adequate polymorphic markers in peanut, bulk segregant analysis is indeed a rapid method to identify markers, particularly genome-wide SNP markers, linked to the target trait. We have identified five SNPs linked to resistance to BW, one of such SNPs was homologous to a RGH. To increase the efficiency of identifying markers linked to the trait, bulk size should be increased. In the present study, the bulk size of 30 individuals might be reduced for identifying tight linked markers. As a smaller bulk is utilized, the frequency of false positives will increase (Michelmore et al. 1991). When less polymorphic markers are available in a given crop species, smaller bulk generating wider target regions allows association studies between markers and the gene underlining the trait of interest but with a more loose association. In contrast, increasing bulk size provides a greater possibility to narrow down the target region to detect tight linkages of markers with target genes, but a large number of genome-wide markers are needed. Increasing the number of individuals in a bulk population may further enhance the accuracy of identified markers linked to disease resistance genes for the target trait, and thus reveal adequate numbers of linked SNP markers.

## Conclusion

Genome-wide markers generated by next-generation sequencing associated with bacterial wilt disease resistance traits using BSA method in this study provided a rapid and effective method for QTL analysis in peanut. As phenotypic data are routinely generated in breeding programs and as the cost for genotyping is constantly decreasing, the identification of markers linked to important traits would be feasible for QTL detection in order to unravel the genetic architecture underlying important traits in peanut. We have identified putative QTL for resistance to bacterial wilt disease, opening up opportunities for future isolation and molecular characterization of QTL using map-based cloning in peanut.

## Electronic supplementary material

Supplementary material 1 (PPTX 917 kb)

Supplementary material 2 (PPTX 844 kb)
