# Supplementary material for: QTL mapping for bacterial wilt resistance in peanut (Arachis hypogaea L.)
Source: Mol Breed. 2016 Jan 30;36:13. doi: 10.1007/s11032-015-0432-0 (PMC4735223; doi:10.1007/s11032-015-0432-0)
Supplement: Supplementary file 1 — Supplementary material 1 (PPTX 917 kb) [file 11032_2015_432_MOESM1_ESM.pptx]

## Slide 1
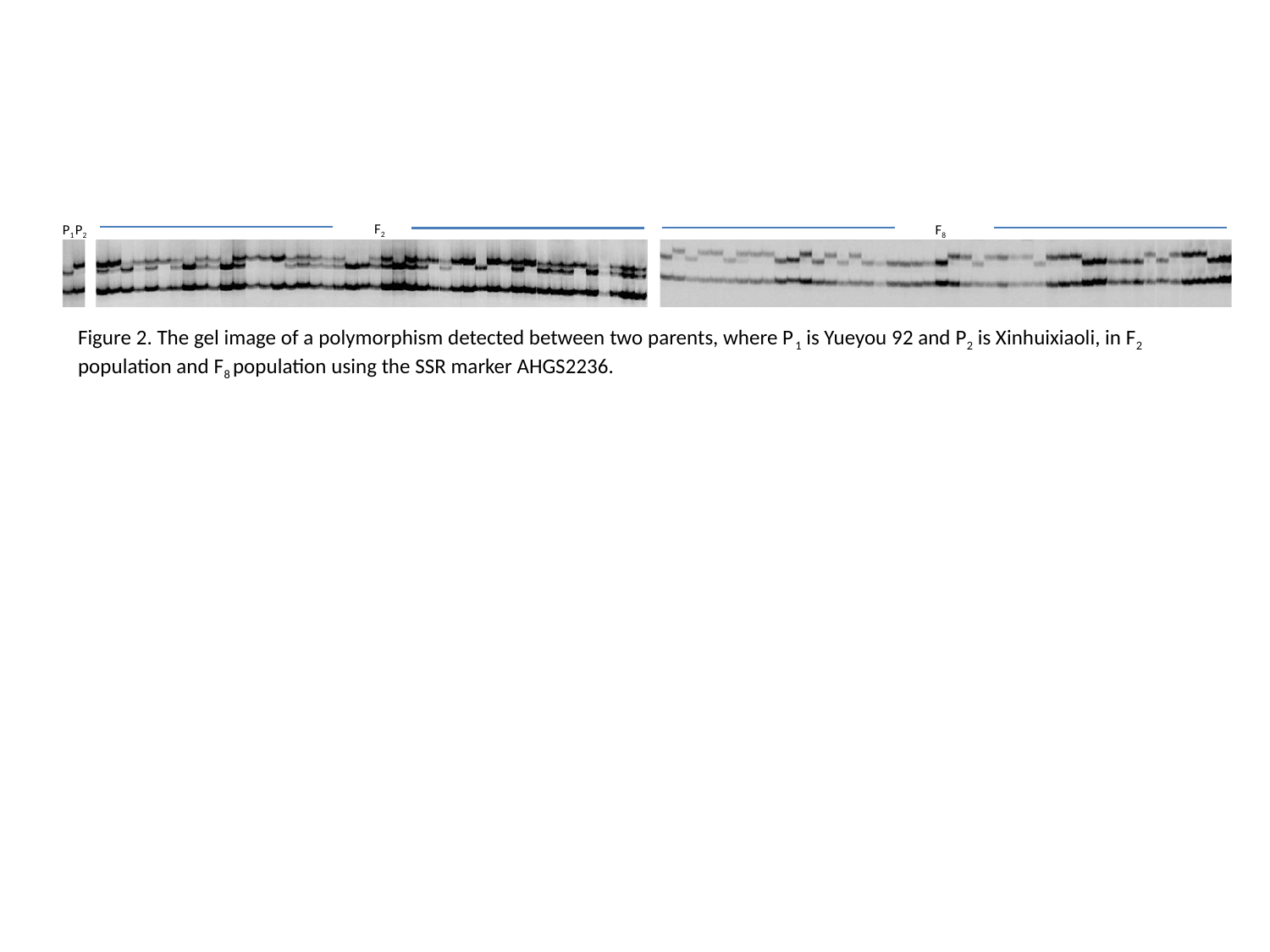

F2
F8
P1
P2
Figure 2. The gel image of a polymorphism detected between two parents, where P1 is Yueyou 92 and P2 is Xinhuixiaoli, in F2 population and F8 population using the SSR marker AHGS2236.
