# Supplementary material for: QTL mapping for bacterial wilt resistance in peanut (Arachis hypogaea L.)
Source: Mol Breed. 2016 Jan 30;36:13. doi: 10.1007/s11032-015-0432-0 (PMC4735223; doi:10.1007/s11032-015-0432-0)
Supplement: Supplementary file 2 — Supplementary material 2 (PPTX 844 kb) [file 11032_2015_432_MOESM2_ESM.pptx]

## Slide 1
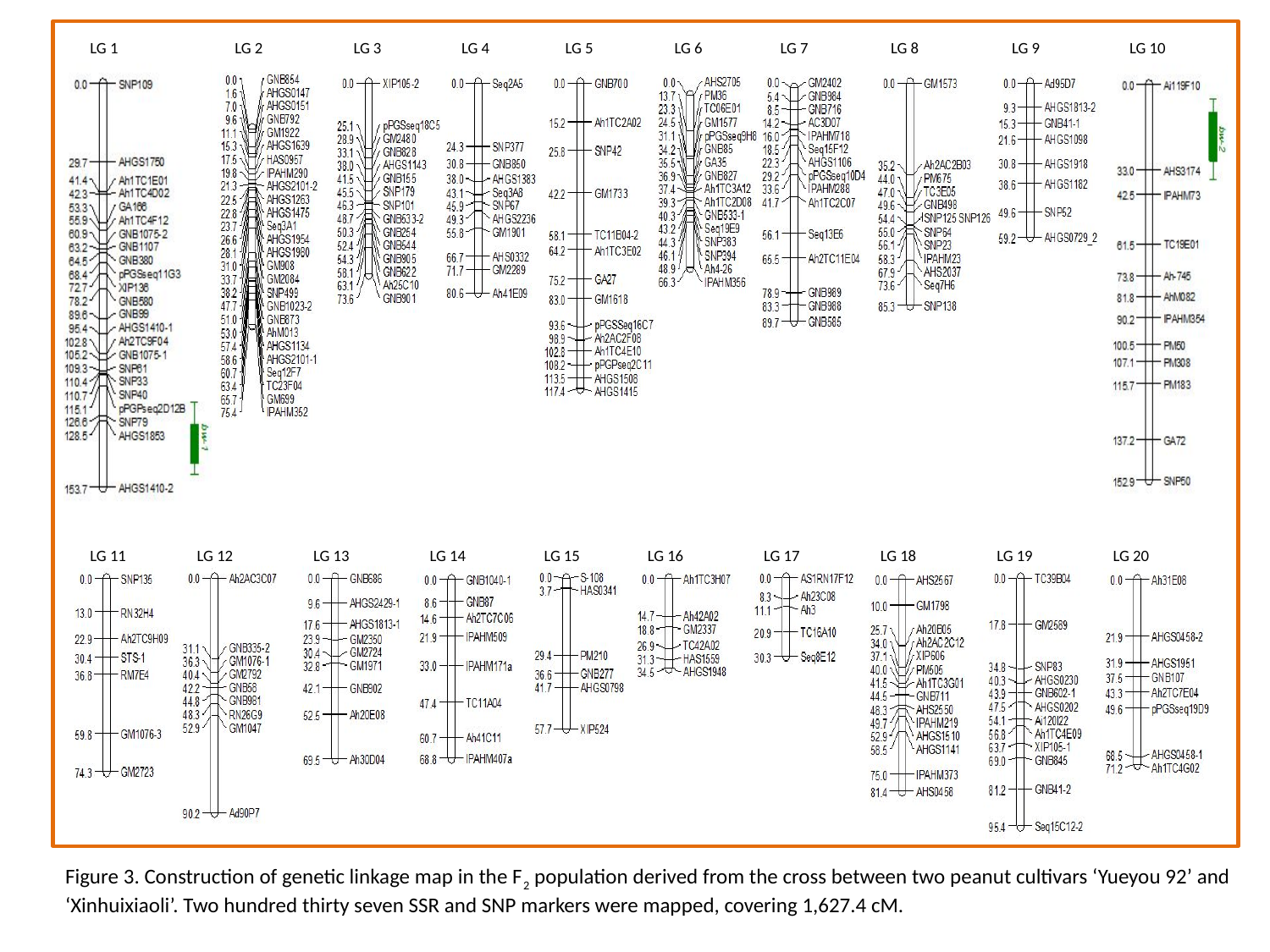

LG 1
LG 2
LG 3
LG 4
LG 5
LG 6
LG 7
LG 8
LG 9
LG 10
LG 11
LG 12
LG 13
LG 14
LG 15
LG 16
LG 17
LG 18
LG 19
LG 20
Figure 3. Construction of genetic linkage map in the F2 population derived from the cross between two peanut cultivars ‘Yueyou 92’ and ‘Xinhuixiaoli’. Two hundred thirty seven SSR and SNP markers were mapped, covering 1,627.4 cM.
